# Effect of the Electric Field on the Distribution Law of Chloride Ions and Microstructure in Concrete with the Addition of Mineral Admixtures

**DOI:** 10.3390/ma12091380

**Published:** 2019-04-28

**Authors:** Xiaoli Xie, Qingge Feng, Zheng Chen, Wei Lu

**Affiliations:** 1 School of Chemistry and Chemical Engineering, Guangxi University, Nanning 530004, China; xiaoli97531@163.com (X.X.); luwei@gxu.edu.cn (W.L.); 2 School of Resources, Environment and Materials, Guangxi University, Nanning 530004, China; 3 Key Laboratory of Disaster Prevention and Structural Safety of China Ministry of Education, School of Civil Engineering and Architecture, Guangxi University, Nanning 530004, China; chenzheng@gxu.edu.cn

**Keywords:** concrete, chloride, electric field, natural immersion, microstructure

## Abstract

Migration testing of chloride under an electric field is a fast and effective method to determine the corrosion resistance of reinforced concrete against chloride. In this study, a series of admixture-involved (fly ash and slag) concrete specimens were produced for an accelerating chloride diffusion test in 3% NaCl solution under an electric field and natural chloride diffusion in 165 g/L NaCl solution under immersion conditions. Then, the chloride profile and pore structure of concretes aged 56 and 91 days were compared to investigate the effect of the electric field on chloride diffusion as well as the microstructure of the concrete. The results showed that, under accelerating electric field conditions, the degree to which chloride refined the internal pore structure of the concrete was weaker than that under natural immersion conditions. The applied electric field changed the pore structure inside the concrete, but it had little effect on the distribution of total, free, and bound chlorides and their mutual relationship. In addition, it is necessary to consider that the electric field effect on chloride migration varies with the concrete mix proportions.

## 1. Introduction

The corrosion of steel in concrete is primarily due to the ingress of the chloride ion, especially in marine environments [1]. The chloride ions in concrete can be divided into two parts: Those inherently contained in raw materials, such as cement, sea sand, and chemical admixture; and those transported from the external environment through pores and micro-cracks in concrete [2]. For chloride ions in the external environment, their ingress into concrete occurs mainly through various erosion mechanisms, such as natural migration and electric-field-assisted migration [3,4,5], and these mechanisms have been widely used in the design of experimental methods for chloride ions to migrate into concrete [6,7] and in determining the migration coefficient of chloride ions in concrete. The results can be used to rate the resistance ability of reinforced concrete against chloride ion corrosion, which is very useful for concrete structural design and material selection. Moreover, these parameters can be used to predict the service life of new and existing buildings.

Natural immersion and electric field acceleration are the two experimental methods for studying the migration of chloride ions in concrete [3,4,5]. Compared with electric field acceleration, natural immersion more closely resembles reality, so the migration coefficient is of higher practical relevance [8]. However, owing to the slow migration rate of chloride ions under a concentration gradient, the experiment is time-consuming. In contrast, when exposed to an external electric field, chloride ions diffuse rapidly owing to the driving force of the electric field, so the migration characteristics of chloride ions inside concrete can be obtained in a short time [9,10,11,12]. However, several studies have questioned the validity of this method by arguing that the application of an external electric field changes the migration property of chloride ions in the concrete, resulting in a migration coefficient far greater than the natural migration coefficient, and changes the microstructure inside the concrete [8,13]. However, some researchers [9,10,11,12,14] studied the immobility of chloride ions under an applied electric field, finding that the applied electric field could accelerate the migration of chloride ions, but had little effect on their distribution. They also found that free chloride ions could be immobilized instantaneously, with the concentration of bound chloride ions increasing with the total chloride ion concentration. Yuan [15] and Spiesz and Brouwers [10] studied the effect of an applied electric field on the adsorption characteristics of chloride ions in concrete by comparing experimental results obtained under natural immersion conditions to those obtained under electric field conditions for samples with the same concrete age at the same temperature. They found that the adsorption isotherms of chloride ions were very similar and concluded that the applied electric field had almost no effect on the adsorption of chloride ions. There remains no consensus on the effects of accelerating electric field conditions and natural immersion conditions on the migration law of chloride ions in concrete and concrete pore structure. Therefore, it is necessary to provide insight into the migration behavior of chloride ions under electric field conditions versus natural immersion conditions, which will provide a theoretical basis to allow a timely design of concrete structures, selection of materials, and prediction of a building’s service life. 

The ASTM C1202 experimental method was developed by the American Society for Testing and Materials for the determination of chloride ion penetrability and is widely used owing to its convenience, time savings, and high repeatability [3]. In this study, the ASTM C1202 method was compared with a natural immersion method to better understand the effect of electric field acceleration and natural immersion as two experimental methods on the migration law of chloride ions and microstructure. Supplementary cementitious materials, such as fly ash and slag, are widely used to improve the concrete pore structure and chloride ion binding ability [16]. Therefore, concrete samples containing different mineral admixtures were prepared in this study and allowed to cure for different periods. The microstructure and hydration product characteristics of the concrete samples were analyzed with mercury intrusion porosimetry (MIP), X-ray diffraction (XRD), differential thermogravimetric analysis (DTG), and scanning electron microscopy (SEM). 

## 2. Raw Materials and Methods

### 2.1. Raw Materials and Concrete Mix Proportions

**Figure 1:**
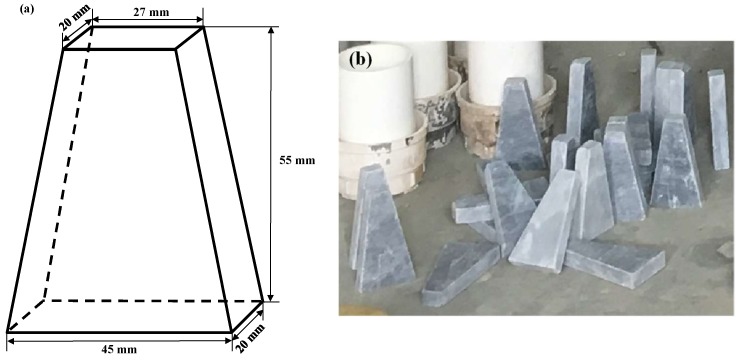
Picture of coarse aggregate: (**a**) schematic diagram and (**b**) real products.

The chemical compositions of Portland cement, fly ash, and slag are shown in Table 1. The fine aggregate was made of local river sand with a fineness modulus of 2.58. To more accurately calculate the concentration of chloride ions in mortar without interference from the aggregate, a specially customized rectangular frustum of granite was used as the coarse aggregate, whose schematic diagram and appearance are shown in Figure 1. The main reason to introduce the customized rectangular frustum of granite was that, under a constant aggregate fraction, the contact area between the mortar and environmental solution at the exposed surface could be enlarged, alleviating the inhibition by the aggregate on the chloride ingress. Further, the longest curing ages employed in this study was 91 days, which was considered insufficient to cause significant disturbance by the active aggregate on properties of the interfacial transition zone (ITZ) in concrete [17]. Another advantage of using aggregates of the same size is to avoid the potential adverse effect of unevenly distributed aggregate properties on the performance of concrete. Deionized water was used as the mixing water and solvent. The water-to-binder ratio (W/B) was 0.45. The replacement level of cement by fly ash or slag was 10% of the weight. The detailed mixing proportions are presented in Table 2, in which the letters, P, F, and G, represent ordinary concrete, fly ash concrete, and slag concrete, respectively.

### 2.2. Sample Preparation and Experimental Procedures

The mortar was agitated following the reported agitation method for high-performance concrete [18]. The agitated mortar was placed in a cylindrical plastic mold with a size of φ 80 mm × 55 mm, and the coarse aggregate was inserted vertically into the mortar with the smaller end facing upwards. The prepared concrete was then cured in an environment with a relative humidity of 50% ± 5% and a temperature of 20 ± 3 °C for one day in the mold. One day later, the concrete was demolded. Next, the concrete products were divided into two groups: One group was allowed to cure under conventional curing conditions (relative humidity of 90% ± 5% and relative temperature of 20 ± 3 °C) for 56 days and 91 days, followed by fast-migration testing of chlorine ions in this group of products under an electric field, and was labeled as group E; the other group, labeled as group N, was allowed to cure for 14 days under conventional curing conditions and then naturally air-dried at 20 ± 1 °C for 7 days, followed by immersion in a 165 g/L NaCl solution for 35 days and 70 days.

### 2.3. Experimental Method

#### 2.3.1. Rapid Chloride Permeability Test

A rapid chloride permeability test was carried out on the concretes in accordance with ASTM Standard C1202 [3] to accelerate the diffusion of chloride ions in the concrete. Each concrete specimen was first saturated in a vacuum water saturation instrument for 24 h and then fixed on a plexiglass cavity. A 3% NaCl solution was used at the cathode, and a 0.3 mol/L NaOH solution was used at the anode. The concrete with the smaller end of the aggregate faced the NaCl solution. Finally, the test was conducted for 6 h continuously under an external electric field of 60 ± 0.5 V.

#### 2.3.2. Natural Immersion Experiment

In order to accelerate the chloride ingress, the concentration of NaCl was designed as 165 g/L (mass fraction 14%) as per NT Build 443 [4], which has been widely used in natural immersion tests. Further, the distribution of the chloride ion concentration could still be fitted based on Fick’s second law [19]. Chloride ions were allowed to migrate through the concrete with the smaller end of the aggregate into the interior of a concrete product that was sealed on all surfaces except for the top surface. Concrete products were placed horizontally in an immersion tank, in which each product was at least 10 mm from any other product and the edge of the immersion tank. The immersion liquid was introduced into the immersion tank to ensure that the liquid completely covered the concrete products, with the product surface at a depth of 20 mm under the liquid surface. The 80 L chloride solution was placed in a container which had a capacity of 85 L, and three samples (dimensions of 80 mm × 55 mm) were immersed in each container. The volumetric ratio of solution to concrete was about 286. Therefore, the total volume of the samples was much less than that of solution, so the stability of concentration of solution as guaranteed. To prevent water evaporation, the immersion box was sealed with a lid, the chloride solution was renewed periodically after every 35 days, and the immersion liquid was agitated and shaken once a week to ensure that the chloride ions diffused uniformly into the concrete.

#### 2.3.3. Preparation of Powder and Bulk Samples 

**Figure 2:**
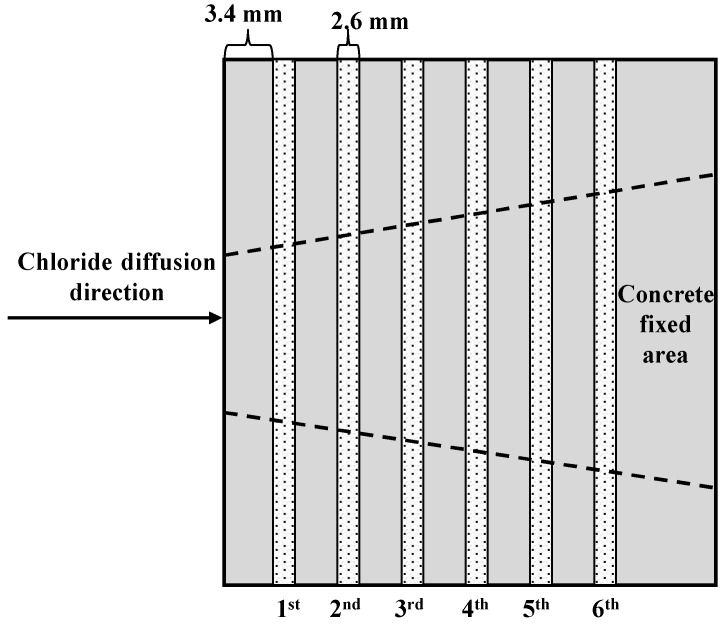
Diagrammatic sketch of the concrete-cutting process.

After the rapid chloride permeability test or natural immersion experiment, the concrete was cut using a 2.6 mm diamond saw blade. To reduce the influence of the heat generated by friction during the cutting process on the chloride ions’ distribution, a low-speed dry cutting process was adopted with a cutting speed of 250 rpm. Each specimen was cut from the end contacting the NaCl solution. Each specimen was cut into 6 layers, and each layer had a thickness of 6 mm. Powder samples and bulk samples were collected during the cutting process. The diagrammatic sketch for the cutting process can be seen in Figure 2. To mitigate the effect of the aggregate size on interfacial transition zone (ITZ) at the exposed side, a sawing depth of 6 mm was selected, and the equivalent depth of the examined chloride concentration based on the collected powders was calculated as 4.7 mm after deducting an additional 3.4 mm thickness from the surface.

#### 2.3.4. Chloride Ion Concentration Measurement

The measurement of the chloride ion concentration was carried out according to the Chinese Standard JTJ 270-98 Testing code of concrete for port and waterway engineering [20,21]. Four grams of the powder sample was divided into two equal portions. One portion was immersed in 100 mL of 5% HNO_3_ solution. The mixture was shaken at 120 rpm for 24 h at 30 °C and then filtered. The concentration of chloride ions in the filtrate was immediately analyzed by titration to obtain the concentration of acid-soluble chloride ions, i.e., the concentration of total chloride ions. The other portion of the powder sample was immersed in 100 mL of distilled water to obtain the concentration of water-soluble chloride ions, i.e., the concentration of free chloride ions. The concentration of bound chloride ions can be calculated by the difference between the concentration of total and free chloride ions.

#### 2.3.5. Analytical Techniques

Mercury intrusion porosimetry (MIP) was employed to characterize the pore structure. An Autopore IV 9500 from Micromeritics was the device employed. The maximum yield pressure was 227 MPa, which enables the measurement of pore diameters from 1,000,000 nm down to 5 nm. Samples with a diameter of 30 mm and length ranging from 20 mm to 30 mm were fabricated and dried in an oven at 105 °C for 24 h before testing.

To determine the crystal phase composition of the prepared powders, X-ray diffraction (XRD) measurement (DX-2700A, DDHaoYuan, Baishan, China) was carried out at room temperature by using a Rigaku D/max diffractometer (Rigaku, Tokyo, Japan) with Cu Kα radiation (*λ* = 1.5406 Å). The maximum angular range of the XRD test conducted in this study was 5 to 90° (2θ), and the final tested range was selected as 10 to 70° (2θ). An accelerating voltage of 40 kV, emission current of 100 mA, and scanning speed of 6 °/min were used.

Thermal properties were determined by thermalgravimetric analysis (DTG-60(H), Shimadzu, Kyoto, Japan). During the experiment, the crucible of alumina pool was used and about 40 mg of sample was filled to the crucible. All the tests were carried out at a heating rate of 10 °C/ min up to 800 °C and a cooling rate of 50 °C/min in a dynamic nitrogen atmosphere.

Surface characterization of tested concrete samples was carried out by scanning electron microscopy (SEM) analysis using a SU8020 (Hitachi High-Technologies, Tokyo, Japan). Samples with a diameter of 30 mm and length ranging from 20 mm to 30 mm were fabricated. The samples were sprayed with gold in high vacuum. The thickness of the coated layer was about 4 nm. An accelerating voltage of 20 kV was used.

## 3. Results and Discussion

### 3.1. Concentration Distribution Patterns of Chloride Ions in Concrete under Different Migration Modes

**Figure 3:**
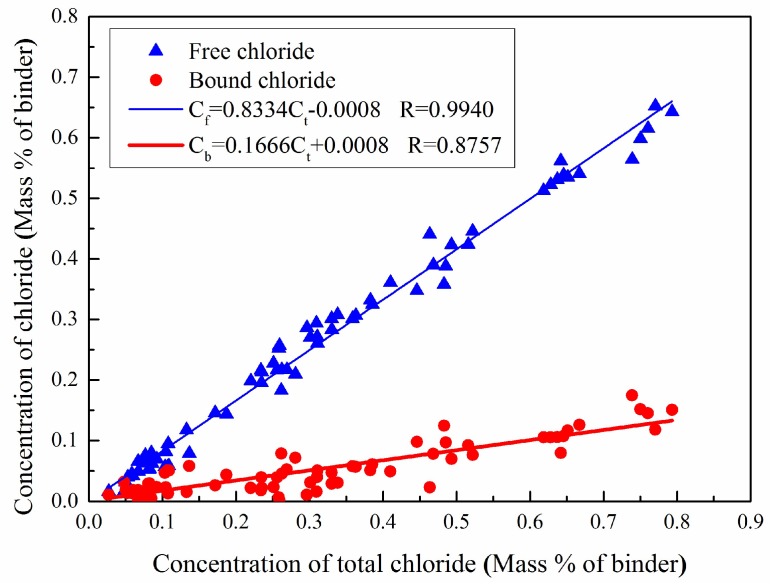
Correlations between free and total chloride ion concentration, and bound and total chloride ion concentration.

Linear, Freundlich, and Langmuir isotherms are commonly used to fit the relationship between the total and free chloride ions [20,22,23]. The concentrations of free chloride ions and bound chloride ions in a group of samples treated under the same condition—natural immersion or electric field acceleration—were linearly regressed against the concentrations of total chloride ions. The results, as plotted in Figure 3, depict that there is a good linear relationship of the total chloride ions to both the free chloride ions and the bound chloride ions in terms of concentration. This result indicates that, although different chloride ion migration modes result in different concentrations of total chloride ions and free chloride ions, the modes have almost no effect on the concentration relationship between chloride ions. This agrees well with the results obtained in other studies [15].

Yuan [15] recognized that the adsorption isotherms of chloride ions were very similar and concluded that the applied electric field had almost no effect on the adsorption of chloride ions. However, this does not mean that the electrical field does not influence chloride binding. The physico-chemical equilibrium would be reached again after the shutdown of the electric field, and extra chloride binding might occur during this period. Therefore, different chloride ion migration modes have little effect on the concentration relationship between the ions. 

### 3.2. Migration Model of Chloride Ions in Concrete based on Fick’s Second Law

Fick’s second law is based on the following three assumptions: (1) The materials are permeable and the internal diffusible medium is homogeneous; (2) there is no chemical reaction, physical absorption, and bonding between materials and the diffused substance; and (3) the diffusion characteristic of materials will not change as the time and concentration vary. The diffusion of chloride ions can be seen to follow Fick’s second law only when the three assumptions mentioned above are satisfied. It is assumed that the migration of chloride ions in concrete under natural immersion conditions is consistent with Fick’s second law [24] as shown by Equation (1), with each variable explained below.

(1) $$\frac{\partial C}{\partial t}=D\frac{\partial^{2}C}{\partial \chi^{2}},$$

where *C* is the concentration of total chloride ions in concrete, *t* is the exposure time of concrete to a chloride ion environment, χ is the distance from the concrete surface; *D* is the migration coefficient of chloride ions.

**Figure 4:**
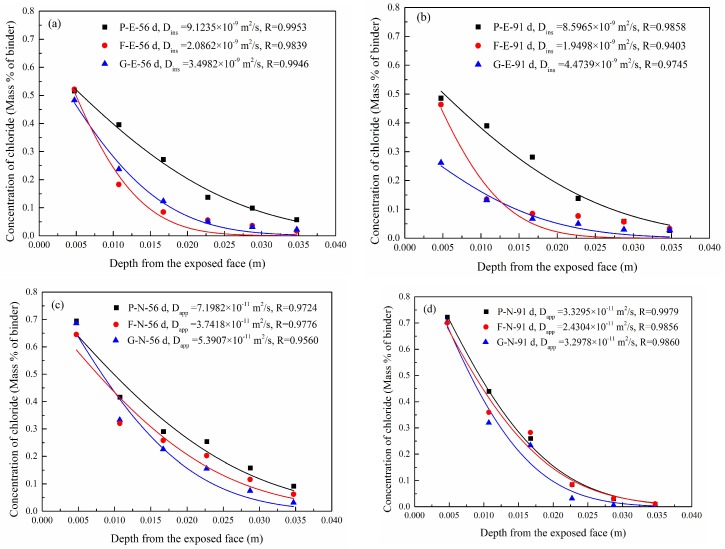
Comparison between molded concentrations and experimentally measured concentrations of chloride ions: (**a**) E-56 d; (**b**) E-91 d; (**c**) N-56 d; (**d**) N-91 d.

Equation (1) can be expressed by Equation (2) with proper initial and boundary conditions, namely, $C(0,t)=C_{S}$, $C(\infty ,t)=C_{0}$, C(χ,0)=0 [25]:

(2) $$C(\chi ,t)=C_{0}+(C_{S}-C_{0})[1-erf(\frac{\chi }{2\sqrt{Dt}})],$$

where C(χ,t) is the concentration of chloride ions at time *t* at a distance, χ, from the concrete surface; *C_0_* and *C_s_* are the initial and surface chloride ion concentrations, respectively; and *erf* is the error function. The concentration distribution of chloride ions in each group of concrete samples was fitted to Equation (2). The migration time under the electric field acceleration condition was 6 h, and the diffusion coefficient obtained here is the instantaneous value, *D_ins_*, whereas the migration times under natural immersion conditions were 35 days and 70 days, and the corresponding diffusion coefficient was the apparent value, *D_app_*. Both the modeled concentrations and the measured concentrations are compared in Figure 4. In Figure 4, the letters, P, F, and G, represent ordinary concrete, fly ash concrete, and slag concrete, respectively. The letters, E and N, represent electric field conditions and natural immersion conditions, respectively. Additionally, 56 d and 91 d denote the curing ages.

As shown in Figure 4, the modeled concentration distribution of chloride ions is consistent with the experimental result with correlation coefficients greater than 0.9403, regardless of natural immersion or electric field acceleration. This indicates that the distribution of chloride ions after electric field acceleration could be fitted as per Fick’s second law. In addition, Figure 4 reveals that the chloride ion concentrations on the concrete surface under natural immersion conditions are greater than those under electric field conditions. The migration of chloride ions in concrete depends largely on the initial concentration, with an increase of the initial concentration leading to an increase of the migration amount and the migration depth of chloride ions [26,27,28]. The chloride ion concentration (14%) in the natural immersion solution of this study was far greater than that (3%) in the solution used for the electric field acceleration experiment, thereby leading to a higher concentration of chloride ions in the naturally immersed concrete.

As mentioned earlier, the diffusion coefficient of chloride ions measured in the electric field (6 h) was the instantaneous value, whereas the other one examined in natural immersion conditions (35 or 70 days) was the apparent value. In order to compare these two diffusion coefficients, the apparent diffusion coefficient should be transformed to the instantaneous diffusion coefficient. As the diffusion coefficient decreases as the concrete age increases, a time-variant diffusion coefficient could be determined based on Equation (3) below [25]:

(3) $$D_{ins}\left(t^{\prime }\right)=D_{0,ins}{\left(\frac{t_{0}^{\prime }}{t^{\prime }}\right)}^{n},$$

where $D_{ins}\left(t^{\prime }\right)$ and $D_{0,ins}$ indicate the instantaneous diffusion coefficient at the moment, $t^{\prime }$, and at a referred moment, $t_{0}^{\prime }$, respectively. n is the instantaneous decay coefficient of the concrete ages.

The instantaneous diffusion coefficient, $D_{ins}\left(t^{\prime }\right)$, reflects the velocity of chloride ions transported in concrete at a specific moment, $t^{\prime }$, whereas the apparent diffusion coefficient, $D_{app}\left(t\right)$, defines the average velocity of chloride ions transported during the whole exposure duration. Therefore, the latter could be expressed as the mean value of the former during the duration, $t=t_{2}^{\prime }-t_{1}^{\prime }$ [29], shown below [30]:

(4) $$D_{app}\left(t\right)=\frac{\int_{t_{1}^{\prime }}^{t_{2}^{\prime }}D_{ins}\left(t^{\prime }\right)dt}{t_{2}^{\prime }-t_{1}^{\prime }}.$$

Then, Equation (4) could be transformed to:

(5) $$D_{app}\left(t\right)=\frac{D_{0,ins}\cdot {\left(t_{0}^{\prime }\right)}^{n}}{1-n}\cdot \left({\left(t_{2}^{\prime }\right)}^{1-n}-{\left(t_{1}^{\prime }\right)}^{1-n}\right),$$

where $t_{1}^{\prime }$ and $t_{2}^{\prime }$ represent the start and end of the exposure, respectively.

Based on the equations above, the apparent diffusion coefficient could be transformed to the instantaneous diffusion coefficient. In this regard, for a certain moment, $t_{ins}^{\prime }$, between $t_{1}^{\prime }$ and $t_{2}^{\prime }$, the magnitude of this instantaneous diffusion coefficient, $D_{ins}$, is equal to the corresponding apparent diffusion coefficient, $D_{app}\left(t_{2}^{\prime }-t_{1}^{\prime }\right)$, namely, $D_{app}\left(t_{2}^{\prime }-t_{1}^{\prime }\right)=D_{ins}\left(t_{ins}^{\prime }\right)$. Note further that $t_{ins}^{\prime }$ could be calculated through the following Equation (6) [31]:

(6) $$t_{ins}^{\prime }=\{\begin{array}{ll} {\left[\frac{\left(1-n\right)\left(t_{2}^{\prime }-t_{1}^{\prime }\right)}{{\left(t_{2}^{\prime }\right)}^{1-n}-{\left(t_{1}^{\prime }\right)}^{1-n}}\right]}^{1/n} & n\neq 0,1\\ \frac{t_{2}^{\prime }-t_{1}^{\prime }}{\ln\left(\frac{t_{2}^{\prime }}{t_{1}^{\prime }}\right)} & n=1 \end{array}.$$

**Figure 5:**
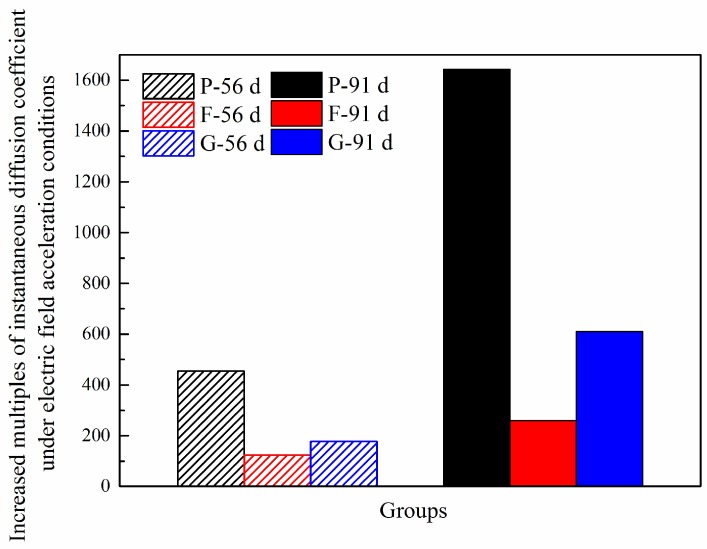
Ratios of the instantaneous diffusion coefficients in concrete under accelerating electric field conditions to those under natural immersion conditions.

Based on the calculated $t_{ins}^{\prime }$ by Equation (6) associated with the corresponding instantaneous diffusion coefficient, $D_{ins}$, the values of n and $D_{0,ins}$ could be determined through fitting. Accordingly, for the specimens exposed to natural immersion, the instantaneous diffusion coefficient at 35 days (aged 56 days) and 70 days (aged 91 days) could be respectively calculated. Then, the corresponding instantaneous diffusion coefficient measured under electric conditions was divided by the one obtained above (in natural immersion conditions). The results are presented in Figure 5, where the letters, P, F, and G, represent ordinary concrete, fly ash concrete, and slag concrete, respectively. Additionally, 56 d and 91 d denote curing ages.

Figure 5 shows that the instantaneous diffusion coefficients are significantly higher under accelerating electric field conditions than under natural immersion conditions, and the ratios increase with the concrete age. In addition, the electric field has a different degree of the accelerating effect on chloride ion migration in concrete with different mix proportions, with the highest accelerating effect appearing in the ordinary concrete, followed by the slag concrete and the fly ash concrete showing the second-highest and weakest accelerating effects, respectively. As the fly ash and slag have more Al_2_O_3_ content than ordinary Portland cement, more hydrated aluminate products are formed in fly ash and slag containing concrete to strengthen the solidification of chloride ions, thus decreasing the diffusion rate [32,33]. Further, the active SiO_2_ incorporated in both fly ash and slag could react with the portlandite (CH) produced by cement hydration to form more C-S-H gel (called secondary hydration reaction), enhancing the physical adsorbability of concrete to chloride ions and refining the internal porosity as well [16]. Hence, the addition of mineral admixtures can mitigate the accelerating effect of an electric field on the migration coefficient of chloride ions. Therefore, when using electric field acceleration to evaluate concrete durability, it is necessary to consider that the same electric field may have varying degrees of an effect on chloride ion migration in concrete with different mix proportions.

### 3.3. Effect of the Applied Electric Field on the Microstructure of Concrete

**Figure 6:**
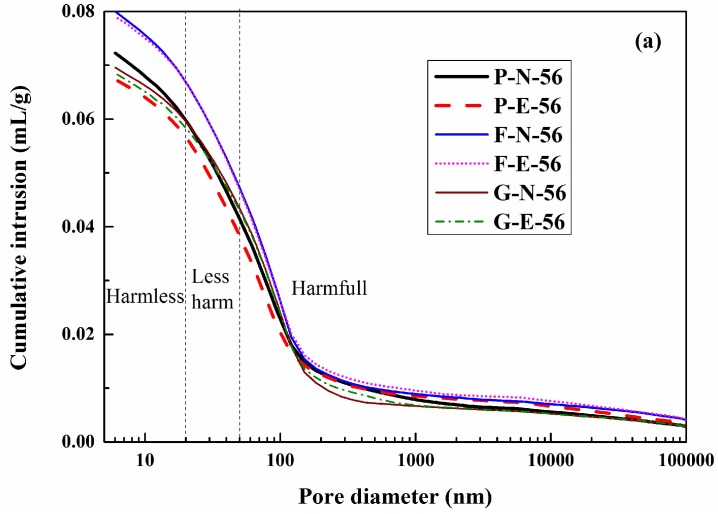

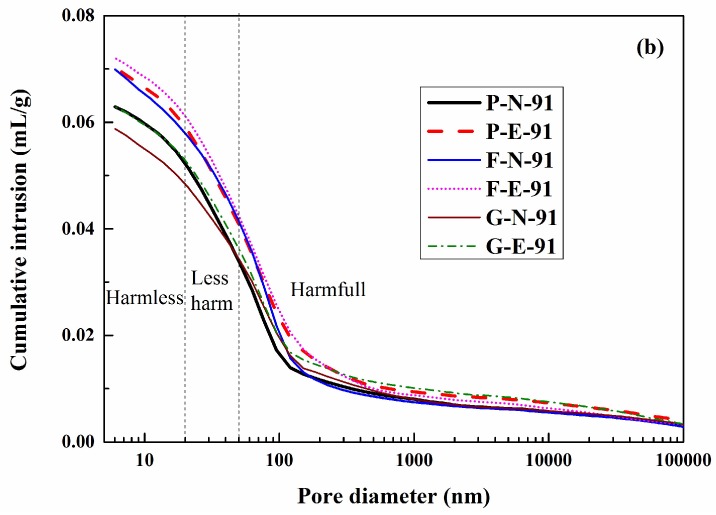
Pore-size distribution of various concretes: (**a**) curing for 56 days, (**b**) curing for 91 days.

The pores in concrete are usually classified into three types: Harmful pores (diameter > 50 nm), less harmful pores (20 nm ≤ diameter ≤ 50 nm), and harmless pores (diameter < 20 nm) [34]. Pore-size distribution of various concretes is shown in Figure 6.

As shown in Figure 6, the concrete under natural immersion conditions has a more refined pore structure than that under accelerating electric field conditions. The pore-size distribution of concrete samples aged 56 days is presented in Figure 6a, which shows that the volume of those pores, leading to no or less harm on concrete, was slightly lower in the case of the accelerating electric field in comparison to natural immersion. However, the volume of harmful pores was higher in the former. As shown in Figure 6b, when the concrete age reaches 91 days, the concrete samples under natural immersion conditions have a smaller pore volume at each pore diameter than those under accelerating electric field conditions. This indicates that, with the aging of concrete, the difference in pore-structure refinement between the samples under natural immersion conditions and those under accelerating electric field conditions is further increased.

In addition, as shown in Figure 6, the difference in the pore-structure distribution between the two migration conditions is large in the ordinary concrete, followed by the slag concrete and the fly ash concrete in order of decreasing difference. Due to the relatively low activity at the early age, fly ash may only show micro-aggregate and morphological effects. However, the activity of fly ash could be triggered as the age reaches 91 days [16]. It could be noted from Figure 6b that even though the fly ash containing concrete registered more pores that cause no or limited harm, the corresponding number of harmful pores decreased significantly. On the other hand, due to the high pozzolanic activity, slag could refine the pore structure of concrete efficiently and improve the chloride resistance, as evident from the refinement of the pore structure shown in Figure 6. However, the quick hydration reaction occurring at the early age may mitigate this influence at 91 days. Thus, the electric field has varying degrees of pore-structure refinement in concrete with different mix proportions.

Many studies have shown [8] that after entering concrete, chloride ions are bound to cause pore-structure refinement. Bound chloride ions consist of chemical binding and physical adsorption chloride ions [35], with the latter primarily referring to chloride ion adsorption by C-S-H gel [36,37] and do not significantly change the concrete pore structure [22,37]. The chemical binding of chloride ions takes place mainly through the formation of Friedel’s salt [36,37]. Friedel’s salt indicates the hydrated chlorine product, which is formed through the reaction between the penetrated chloride ions and hydration products of C_3_A in concrete. This product has a large volume and can refine the pore structure of concrete [8,13]. Therefore, chloride ingress should refine the pore structure of concrete.

As shown by the analysis results of the chloride ion concentrations in Section 3.2, the concentrations in concrete under natural immersion conditions are greater than those under accelerating electric field conditions. Moreover, chloride ions under natural immersion conditions have a longer time in contact with hydration products, such as aluminate, so natural immersion conditions are more conducive to the formation of Friedel’s salt [9,22]. This explains why the pore structure in concrete is more refined under natural immersion conditions than under accelerating electric field conditions. With the increase of the concrete age from 56 days to 91 days, the duration for the natural immersion of concrete in the chloride ion solution increases from 35 days to 70 days, which promotes chloride ion concentrations in the naturally immersed concrete. In contrast, owing to a higher degree of concrete hydration under accelerating electric field conditions, fewer chloride ions migrate into the concrete, making an even larger difference in the formation of Friedel’s salt between the two migration conditions. As a result, by the time the concrete has aged 91 days, the difference in the pore distribution inside the concrete between the two migration modes is further increased, which explains why the ratio of the chloride ion migration coefficient under accelerating electric field conditions to that under natural immersion conditions increases with the concrete age.

The addition of fly ash and slag, both containing a higher Al_2_O_3_ content, into ordinary concrete can form more aluminate hydration products, making it more likely that Friedel’s salt will form between the aluminate hydration products and the chloride ions. Fly ash is higher than slag in Al_2_O_3_ content, and both are higher than ordinary Portland cement. Therefore, when placed under the same conditions of electric field acceleration, fly ash concrete has a higher probability of forming Friedel’s salt than slag concrete, and both have a higher probability than ordinary concrete. Under natural immersion conditions, given the high chloride ion concentration in the solution and the long contact time with the aluminate hydration product, a higher Friedel’s salt content can be formed in concrete with various mix proportions [9,22], leading to an improvement of the pore structure inside the concrete [8]. Besides, the fly ash and slag can generate a positive effect on the pore structure of concrete and therefore improve the corresponding chloride resistance through three effects, i.e., morphological, activated, and micro-aggregate effects [33]. Therefore, the difference in the pore-structure distribution between the two migration conditions is large in ordinary concrete, followed by the slag concrete and the fly ash concrete in order of decreasing difference. This results in a difference in the accelerating effect of the electric field on chloride ion migration between different concrete mix proportions. 

### 3.4. Results and Analysis of XRD, DTG, and SEM Measurements

**Figure 7:**
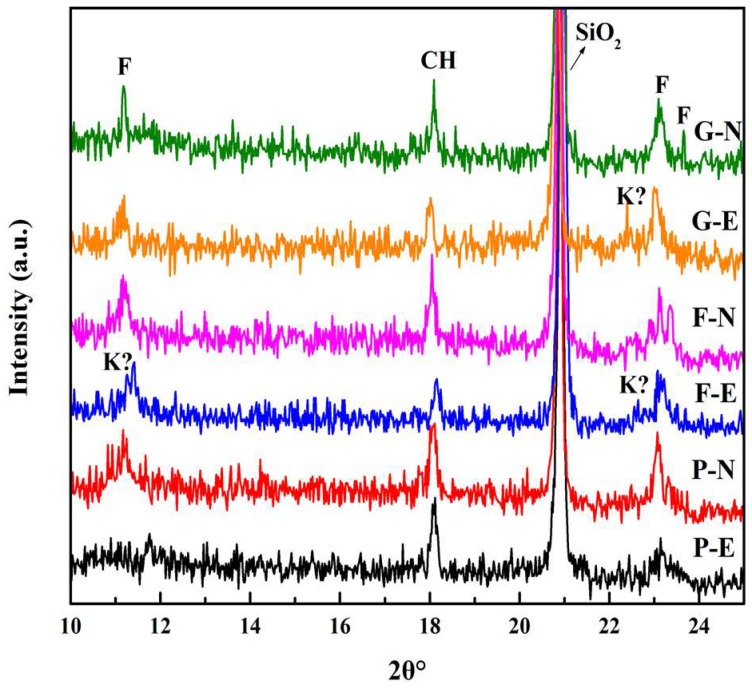
X-ray diffraction (XRD) diffractograms for the concretes. F = Friedel’s salt, K? = Kezul’s salt?

**Figure 8:**
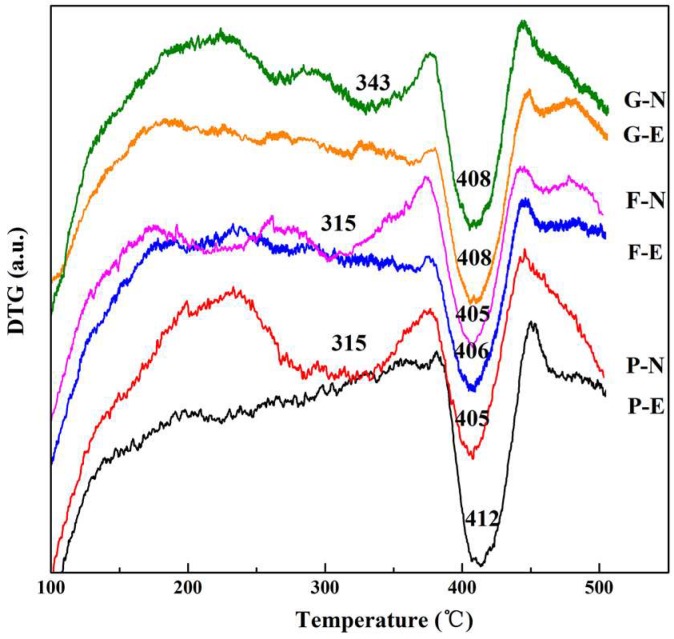
Differential thermogravimetric analysis (DTG) for Friedel’s salt in the concretes.

**Figure 9:**
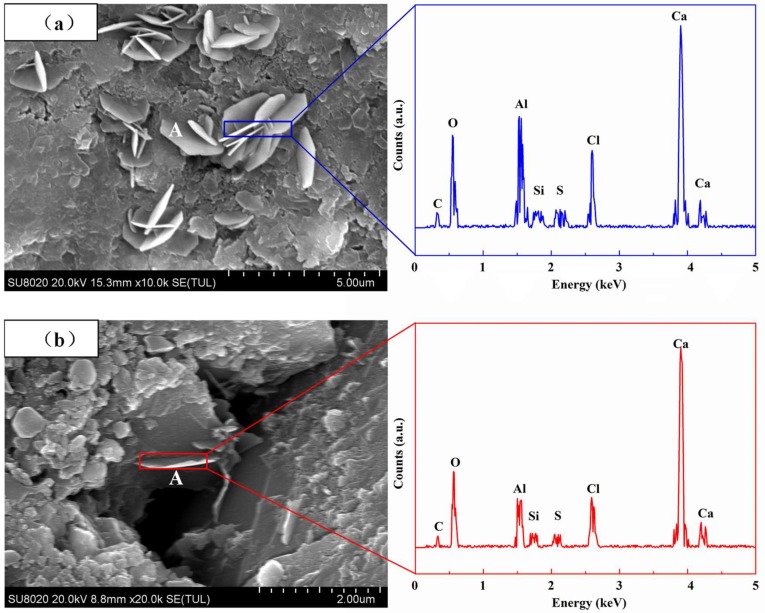
Scanning electron microscopy (SEM) diagram of concrete under different migration conditions: (**a**) F-N-91 day; (**b**) F-E-91 day.

X-ray diffraction, differential thermogravimetric analysis (DTG), and scanning electron microscopy (SEM) measurements were performed on the 91-day concrete samples treated with two conditions of chloride ion migration, and the measurement results are shown in Figure 7, Figure 8 and Figure 9. In addition, the area of each diffraction peak could be calculated through fitting the XRD spectrum. The areas of CH or Friedel’s salt peak in variously produced concrete were added together and then used to calculate the relative variational ratio, *R*, as per Equation (7). The results are shown in Table 3.

(7) $$R=\frac{m_{1}-m_{0}}{m_{0}},$$

where *m*_0_ is the area proportion of CH or Friedel’s salt peak registered by the reference group, P-N; *m*_1_ indicates the area proportion of CH or Friedel’s salt peak comprised of each examined group (P-E/F-N/F-E/G-N/G-E). The DTG curve was obtained by taking the derivative of the tested thermalgravimetric profile, and the various peaks represented the mass variation in terms of each hydrated product right before and after the thermal decomposition. The mass loss of the examined hydrated product could be determined by calculating the peak area. Eventually, together with the total mass, the mass percentage of each hydrated product was therefore determined—see Table 4.

As shown in both Figure 7 and Table 3, Friedel’s salt could be found under both diffusion conditions (electric field and natural immersion). However, the peak intensity, sharpness, and area of Friedel’s salt are all much lower under the electric field, especially for ordinary concrete. Further, the F-N and G-N series register the higher proportion of Friedel’s salt, but a lower CH proportion as compared to the P-N case. To further confirm the XRD peaks assigned to Friedel’s salt, DTG measurement was performed, and the results are shown in Figure 8 and Table 4. Friedel’s salt undergoes endothermic decomposition at 310 to 380 °C. CH undergoes endothermic decomposition at 450 °C [32,38]. As shown in Figure 8, there is a peak showing significant change due to the decomposition of Friedel’s salt in concrete under natural immersion conditions. This observation confirms that the F peak in the XRD patterns of concrete treated with natural immersion conditions is assigned to Friedel’s salt, whereas there is no obvious weight change at 310 to 380 °C for concrete treated with electric field conditions, especially for ordinary concrete. This suggests that, under electric field conditions, Friedel’s salt is low in content or has been partially decomposed. Further, as shown in Table 4, the CH contents in the F-N and G-N series are lower than P-N, which has good agreement with the XRD results. On the other hand, a higher content of Friedel’s salt was found in the P-N case as compared to F-N and G-N. This may be attributed to the fact that the P-N set had a wider peak in terms of mass variation, showing that Friedel’s salt can be present as a solid solution (anionic chloride ions replaced partly or fully by OH^-^ or as Kezul’s salt) [39]. Therefore, the Friedel’s salt content calculated here should be the content of a solid solution.

Again, the peak intensity, sharpness, and area of Friedel’s salt were much lower under electric field, indicating a worse crystallinity. In addition, some peaks around the peak of Friedel’s salt might belong to Kezul’s salt (formed by the decomposition of Friedel’s salt) [40,41]. This is primarily attributed to the rapid ingress of chloride ions into the concrete under accelerating electric field conditions, resulting in the chloride ions having a short contact time with the hydration products of C_3_A. Moreover, the low chloride ion concentration in the immersion solution under accelerating electric field conditions is not conducive to the formation of Friedel’s salt [9,22,42]. The pH of the concrete pore solution is changed under electric field conditions [43,44], which affects the stability of the previously formed Friedel’s salt [12,45], resulting in partial decomposition of Friedel’s salt to form Kezul’s salt under electric field conditions [40,41]. Besides, the XRD results revealed that the peak intensity, sharpness, and area of CH were much lower under the electric field. Also, as seen from both Table 3 and Table 4, the proportion of CH was lower under the electric field as compared to the natural immersion conditions. This may be attributed to the decomposition of a part of CH under the electric field. Therefore, the stability of Friedel’s salt and CH under electric field conditions are not as good as those in naturally immersed concrete, resulting in a weaker degree of pore-structure refinement under electric field conditions than under natural immersion conditions. 

Seen from the XRD and DTG results, for both fly ash and slag, the corresponding samples aged for 91 days registered a lower CH content as compared to ordinary concrete, and this should mainly be attributed to the pozzolanic activity. Besides, the electric field was found to have the potential to yield the dissolution of CH and Friedel’s salt, leading to a negative influence on the compactness of the pore structure of concrete. However, the pore structure was refined in the presence of fly ash and slag due to the formation of C-S-H gel through pozzolanic reactions. Furthermore, the dissolution of CH under the electric field may cause less of an effect on the pore structure than ordinary concrete, because a part of CH has already been consumed to form C-S-H gel prior to dissolution.

Figure 9 depicts the SEM images of fly ash concrete. Given that Friedel’s salt is a hexagonal, flake like structure with a size of 2 to 3 μm, CH may also be hexagonal, but is much larger than Friedel’s salt, and C_3_A is hexagonal with a size below 1 μm and is smaller than Friedel’s salt [32], it is deduced that object A in Figure 9 may be Friedel’s salt. To further confirm this deduction, energy-dispersive X-ray spectroscopy (EDS) analysis was performed on object A, and the results are shown in Figure 9. The EDS analysis results indicate that the main compositional elements of object A are Ca, Al, O, and Cl, in agreement with those of Friedel’s salt [13]. This confirms that object A is Friedel’s salt. It is difficult to discern Friedel’s salt in concrete treated with electric field conditions; it is small in size and dispersed in distribution, in proximity to the hydration products of C_3_A, making it difficult to identify. This is mainly because, under electric field conditions, chloride ions take a short time to react with C_3_A hydration products, making the formed Friedel’s salt small in size and dispersed in distribution. Moreover, the chloride ion concentration is low, which is not conducive to the formation of Friedel’s salt and may even result in the dissolution of Friedel’s salt to form Kezul’s salt [41]. In contrast, it is easier to discern Friedel’s salt in concrete treated with natural immersion conditions, and Friedel’s salt is larger with clear edges. This is mainly because, under natural immersion conditions, chloride ions have a longer contact time with C_3_A hydration products, which facilitates the formation of large flake-shaped Friedel’s salt. Moreover, the chloride ion concentration is high, which is beneficial to the growth of Friedel’s salt and prevents it from dissolving [40,41]. Therefore, there are significant differences in the size and morphology of Friedel’s salt between the different chloride migration modes.

## 4. Conclusion 

The distribution law of chloride ions and microstructural characteristics in concrete treated with two types of migration conditions, electric field conditions versus natural immersion conditions, were compared, giving insight into the effects of different chloride migration modes on the distribution law and microstructural characteristics. The main conclusions are as follows:

(1) Chloride ion ingress into concrete can change the pore structure of the concrete. Compared with accelerating electric field conditions, chloride ions provide a greater degree of pore-structure refinement in concrete under natural immersion conditions.
(2) Whether under natural immersion conditions or under accelerating electric field conditions, the concentrations of total chloride ions have a significant linear relationship with those of free chloride ions and those of bound chloride ions. The applied electric field changes the concentration of chloride ions migrating into the concrete and their ingress rate, thereby changing the morphology and crystallinity of Friedel’s salt. However, the electric field has little effect on the distribution of total, free, and bound chloride ions and their mutual relationship.
(3) The concentration distribution of chloride ions in concrete under accelerating electric field conditions can be fitted as per Fick’s second law. The electric field has varying degrees of an accelerating effect on chloride ion migration in concrete with different mix proportions. In particular, the accelerating effect is greater in ordinary concrete than in slag concrete and is weakest in fly ash concrete. This was mainly attributed to the fact that the electric field results in the dissolution of CH and Friedel’s salt, leading to a negative effect on the compactness of the pore structure of concrete. However, the pore structure is refined in the presence of fly ash and slag due to the formation of C-S-H gel through pozzolanic reactions. Furthermore, the dissolution of CH under an electric field may have less of an effect on the pore structure than ordinary concrete, because a part of CH has already been consumed to form C-S-H gel prior to dissolution. Therefore, when evaluating concrete durability by accelerating chloride ion migration under an electric field, it is necessary to consider the varying degrees of the electric field effect on chloride ion migration in concrete with different mix proportions.

## Figures and Tables

**Table 1:** Chemical composition of cement, fly ash, and slag (%).

Compositions	SiO_2_	Al_2_O_3_	Fe_2_O_3_	CaO	MgO	SO_3_	f-CaO	K_2_O + Na_2_O
Cement	22.92	4.82	3.46	64.48	1.93	0.64	0.52	0.27
Fly ash	52.15	26.19	11.80	3.81	1.34	0.89	-	0.84
Slag	27.60	14.99	0.61	35.76	10.29	0.41	-	0.60

**Table 2:** Mixture proportion of mortar.

Groups	W/C	Mix Proportions (kg/m^3^)				
		Water	Cement	Fly ash	Slag	Sand
P	0.45	180	400	0	0	800
F	0.45	180	360	40	0	800
G	0.45	180	360	0	40	800

**Table 3:** Rate of increase or decrease in the relative composition of crystalline compounds in each concrete with respect to P-N.

Groups	Rate of Increase/Decrease (%)			
	Natural Immersion Conditions		Electric Field Conditions	
	CH	Friedel’s Salt	CH	Friedel’s Salt
P	Reference	Reference	−4.56	−62.81
F	−12.77	33.06	−20.26	−31.13
G	−6.57	25.90	−19.34	−17.44

**Table 4:** Contents of CH and Friedel’s salt in each concrete.

Groups	Natural Immersion Conditions		Electric Field Conditions	
	CH	Friedel’s Salt	CH	Friedel’s Salt
P (%)	0.382	0.265	0.252	0
F (%)	0.308	0.237	0.186	0.165
G (%)	0.313	0.186	0.192	0.137
